# Use of a Conversational Agent for Training Mental Health Professionals in Suicide Safety Planning: Pilot Feasibility and Acceptability Study

**DOI:** 10.2196/88440

**Published:** 2026-06-30

**Authors:** Bénédicte Nobile, Zohar Elyoseph, Elia Gourguechonbuot, Josselin Guyodo, Jordi Garcia, Inbar Levkovich, Emilie Olie, Yuval Haber, Yossi Levi-Belz, Philippe Courtet

**Affiliations:** 1 University of Haifa, 199 Aba Khoushy Ave, Mount Carmel, Haifa, 3498838, Israel, 972 555009984; 2 The Lior Tsfaty Center for Suicide and Mental Pain Studies, University of Haifa, Haifa, Israel; 3 Centre Hospitalier Universitaire de Montpellier, Montpellier, Occitanie, France; 4 Tel Hai Academic College, Upper Galilee, Northern District, Israel; 5 The Program for Hermeneutics and Culture, Interdisciplinary Studies Unit, Bar-Ilan University, Ramat Gan, Israel

**Keywords:** safety plan, suicide, artificial intelligence, large language models, clinical formation

## Abstract

**Background:**

Safety planning is recognized as one of the most effective interventions for reducing suicidal behaviors. The quality of safety plans strongly depends on professional training, and traditional methods, such as role-playing, are time-consuming and offer limited opportunities for repetition across diverse patient profiles. Generative artificial intelligence (GenAI) may provide innovative solutions by offering accessible, flexible, and realistic training environments.

**Objective:**

This pilot study aimed to evaluate the acceptability and feasibility of a GenAI-based simulator designed to train mental health professionals in safety planning.

**Methods:**

Twenty nurses and nursing assistants from psychiatric units in a French university hospital participated in a pre-post, single-session evaluation. After self-rating their ability, competence, and willingness to manage patients experiencing suicidal ideation, participants interacted individually with the text-based simulator for 20 minutes to perform a safety plan with a chatbot, then completed postsimulation acceptability items, and open-ended feedback. Composite scores were computed: acceptability (eg, helpfulness; 0‐40), realism (eg, looking like real interaction with patient; 0‐20), and challenge (eg, emotional challenge; 0‐30). Pre-post changes were tested (Wilcoxon signed-rank test), and age-group comparisons were performed.

**Results:**

Acceptability was high (mean 31.9/40, SD 5.3; median 32, IQR 7), realism moderate-to-high (mean 15.1/20, SD 4.1; median 15, IQR 5.25), and challenge manageable (mean 17.0/30, SD 8; median 18, IQR 12.5). Participants rated usefulness (mean 7.65/10, SD 1.57; median 8, IQR 1.57), perceived learning (mean 7.6/10, SD 1.79; median 8, IQR 2), recommendation to use the chatbot for training (mean 8.3/10, SD 1.59; median 9, IQR 2.25), and feedback quality (mean 8.35/10, SD 1.27; median 8.5, IQR 1.25) favorably. Willingness to actively manage patients experiencing suicidal ideation significantly increased postsimulation (*P=*.03). Younger participants reported higher acceptability (*P=*.04) and realism (*P=*.03). Participants reported minimal concerns regarding the simulator’s use.

**Conclusions:**

This pilot study demonstrates that a GenAI-based simulator for safety planning is feasible and highly acceptable among experienced mental health professionals. The findings are promising and warrant larger, controlled trials to assess impacts on training effectiveness and patient outcomes.

## Introduction

Every 40 seconds, one person dies by suicide worldwide [1]. Currently, suicide is the 17th leading cause of death across the lifespan and the fourth leading cause of death among people aged 15 to 29 years [1], and there are 20 to 30 times more suicide attempts [2]. Moreover, these alarming numbers may even be underestimated due to stigma and misclassification [1]. As suicide attempts are the strongest risk factor for future death by suicide as well as a major risk factor of reattempts, it is imperative to develop tools to reduce the risk of suicide attempt and reattempt [3,4]. Among the currently available interventions, safety planning is one of the most widely used and has proven highly effective in reducing suicidal behaviors [5,6]. Safety planning is a brief intervention that aims to help patients during a suicidal crisis through a set of steps designed to reduce the likelihood of engaging in suicidal behavior. The steps include identifying (1) warning signs or triggers that indicate suicidal ideation is likely to occur, (2) internal coping strategies (eg, distracting activities) to use when those triggers occur or when experiencing suicidal ideation, (3) social contacts or locations that may provide distraction, (4) supportive contacts (who can provide assistance), (5) emergency resources (eg, therapist phone numbers, hotlines), and (6) measures to ensure a safe home environment that minimize the patient’s ability to act on suicidal thoughts or urges (ie, reducing access to lethal means) [5,6]. For example, in a recent meta-analysis including 3536 patients, those receiving safety planning intervention had a 43% lower risk of suicidal behaviors compared to those who did not receive the intervention [7]. Numerous studies have already demonstrated the effectiveness of safety planning, not only in reducing the risk of suicidal behaviors but also in lowering hospitalization rates and increasing patient engagement in care [7-9]. Consequently, both the Suicide Prevention Resource Center and the Joint Commission recommend safety planning as a standard of care for individuals identified as at risk for suicidal behaviors [5,10].

Usually, a safety plan is developed collaboratively by the patient and a mental health care professional. Therefore, mental health professionals need to be trained to correctly implement safety planning. This training is particularly important since the quality of the safety plan has a significant impact on its effectiveness [5,11]. Indeed, a recent study conducted on 200 high-risk patients found that high-quality safety planning was associated with fewer psychiatric hospitalizations [11]. The quality of safety planning is defined by its completeness (eg, all 6 steps are fulfilled and detailed) and by the extent to which each item reflects multiple, specific, and personalized responses. Furthermore, the collaborative process between the patient and the provider is also crucial, as the latter must provide all relevant information to ensure that the safety plan can be effectively used by the patient. In addition, a large proportion of mental health professionals who had received specific training reported the need for further training [5].

Different types of training exist, but one of the most commonly used approaches combines theoretical instruction with role-playing exercises (ie, the trainee acts as the provider, while the trainer takes the role of the patient). A major limitation of this method is the amount of time it requires. Role-playing exercises cannot last very long due to the trainer’s limited availability, and the number of times these exercises can be repeated with trainees is therefore restricted. However, the greater the number of practice repetitions—particularly when involving a wide variety of patient profiles—the more skilled and confident the professional becomes in developing safety plans, regardless of the type of patient encountered. It is therefore necessary to find more efficient ways to train mental health professionals in safety planning. In the era of generative artificial intelligence (GenAI), this technology may be particularly useful for such training purposes. Indeed, growing evidence has demonstrated the potential benefits of GenAI in the field of mental health care, as well as in the domain of suicidal behaviors [12-17]. More specifically, GenAI can be used for the training of health care professionals. For example, in a recent study, a GenAI-based simulator was created to train mental health professionals in the assessment of suicidal ideation and showed promising results [16].

In this context, a GenAI-based simulator was developed to train mental health care professionals in conducting safety plans with patients. In this simulator, the system takes on the role of a patient through a vignette designed by our team, with the possibility of using different vignettes to represent various patient profiles. After completing the safety plan with the simulated patient in writing, the trainee receives feedback highlighting strengths as well as areas for improvement. The aim of our study was, therefore, to assess the acceptability and feasibility of using such a tool among mental health professionals specialized in the care of patients experiencing suicidal ideation.

## Methods

### Participants

Twenty health care professionals were recruited for this study, including nurses and nursing assistants working in the psychiatric units of the University Hospital of Montpellier (CHU Lapeyronie, France). Participants were working in one of the following services: (1) 19-bed acute care unit specialized in the management of patients experiencing suicidal ideation, (2) 21-bed inpatient unit specialized in the management of patients with mood disorders, or (3) outpatient team providing short-term and close follow-up care for patients experiencing suicidal ideation discharged from the emergency unit. Participants were nurses and nursing assistants, as these professionals are primarily responsible for conducting safety planning in the participating units. No formal inclusion or exclusion criteria were applied beyond working in these units and regularly performing safety plans, and all eligible volunteers were included. Although this study focused on these professionals, the simulator is designed to be applicable to a broader range of health care providers involved in suicide prevention.

### Ethical Considerations

The institutional research office of the University Hospital of Montpellier determined that this study did not fall under the scope of the French Jardé Law (Article R1121-1) [18] and therefore did not require ethics committee approval. All procedures complied with General Data Protection Regulation requirements. Participants received written information and provided written informed consent. Data were pseudonymized and stored on secure institutional servers.

### GenAI-Based Simulator

The entire system is built upon a sophisticated prompt architecture designed for Anthropic’s Claude 3.5 Sonnet V2 model and was deployed to participants via the PMFM artificial intelligence (AI) platform. The methodology is exclusively based on advanced prompt engineering, with no model fine-tuning.

### Simulator Design and Architecture

The simulator is designed around a dual-persona, 2-stage paradigm to provide a holistic learning experience encompassing both practice and reflection.

1. Stage 1: clinical role-play simulation. In this stage, the AI embodies the persona of “Sophie Moreau,” a 30-year-old patient recently hospitalized following an aborted suicide attempt. Participants engage in a real-time, text-based conversation with Sophie, with the explicit goal of collaboratively constructing a safety plan, which streamlines the standard 5-step plan by consolidating the steps for contacting personal supports (family or friends) and professional services into a single “seeking help” step.
2. Stage 2: expert feedback and debriefing. Upon the completion of the role-play, the AI transitions to its second persona, “Camille,” a clinical psychologist. In this role, the AI analyzes the entire interaction transcript and provides structured, critical feedback based on a predefined 11-point rubric and scoring system.

The simulator’s architecture integrates 2 foundational knowledge sources provided as appendices in Multimedia Appendices 1-4 within the core prompt: a clinical framework for the 5 steps of safety planning and a comprehensive patient profile for Sophie Moreau.

The clinical framework is based on the Safety Planning Intervention [6]. The 5-step structure served as the backbone of both the simulation interaction and the feedback evaluation. The safety plan was constructed collaboratively within the text-based conversation—participants worked through each step sequentially with the simulated patient, and no separate paper or digital template was used. The resulting plan was contained within the conversation transcript; while the platform supports transcript storage and export, conversations in this study were conducted anonymously to ensure participant privacy (see Multimedia Appendix 1 for details). While other approaches to safety planning exist, such as Crisis Response Planning [19], the Safety Planning Intervention was selected given its widespread adoption in clinical practice and in the participating hospital units. The technical implementation of the simulator, including model selection, platform deployment, and prompt engineering strategies, is described in detail in Multimedia Appendix 1.

### Procedure

Three training sessions were conducted between February and May 2025—1 per psychiatric unit—with a single session attended by all participants from each unit (n=10, n=4, and n=6 participants, respectively). Each participant took part in only 1 session. Each session lasted approximately 1 hour and was jointly facilitated by two trainers: (1) an advanced practice nurse specialized in the management of patients experiencing suicidal ideation and responsible for safety plan training within the psychiatric emergency and postemergency department of the University Hospital of Montpellier and (2) a researcher specialized in suicide behavior research, with expertise in the use of AI for suicide prevention. The facilitators were responsible for introducing the tool, providing instructions, and assisting participants in case of technical difficulties but did not intervene during the simulation itself. Each session lasted approximately 1 hour, including the introduction (~10 min), completion of the presimulation questionnaire (~10 min), individual interaction with the simulator (~20 minutes to complete the safety plan and ~10 minutes of automated debriefing), and completion of the postsimulation questionnaire (~10 min). The debriefing was automated and provided directly by the simulator at the end of the interaction. At the beginning of each session, the trainers introduced the simulator and explained its functioning to the participants. Participants then completed the first part of a self-administered questionnaire assessing their background (eg, gender, age, years of clinical experience, prior training in safety planning) and their perceptions regarding their ability, competence, and preparedness to conduct safety planning (ie, Likert-type scales).

After this initial assessment, participants interacted individually with the GenAI-based simulator for approximately 20 minutes. Although shorter than the time usually required to complete a full safety plan in clinical practice, this duration was chosen to accommodate the organizational constraints of clinical services. The objective of this limited interaction was primarily to evaluate the acceptability and feasibility of using such a tool in a population of mental health care professionals specialized in the care of patients experiencing suicidal ideation. Following the simulation, participants completed the second part of the questionnaire (described below), which focused on their experience with the simulator, perceived usefulness for training, quality of feedback received, perceived realism compared to clinical practice, and suggestions for improvement.

### Measures

Data were collected using a self-administered questionnaire specifically designed for this study. The questionnaire consisted of three sections:

1. Sociodemographic and professional characteristics: This section collected information on gender, age, years of clinical experience in mental health, professional role (nurse or nursing assistant), and prior training in safety planning. Participants were also asked about any previous exposure to AI in psychiatric care.
2. Presimulation assessment: Before interacting with the simulator, participants rated their perceptions regarding safety planning on Likert-type scales (0‐10). Items assessed their perceived ability, competence, and resources to develop a safety plan, as well as their willingness to care for patients experiencing suicidal ideation.
3. Postsimulation assessment: After completing the simulation, participants rated their experience with the GenAI-based simulator using Likert-type scales (0‐10). Items included perceived usefulness of the experience for future safety planning, perceived learning, recommendation of such training to other professionals, self-perceived ability, competence, and resources to conduct safety planning, interest in working with patients experiencing suicidal ideation, perceived quality of feedback, levels of discomfort, cognitive and emotional challenge, realism of the interaction, and similarity to real clinical practice. Additional open-ended questions explored participants’ subjective experiences, perceived advantages and limitations of the training, and suggestions for improvement.

### Composite Scores

In addition to single-item analyses, three composite scores were constructed to provide a global evaluation of the simulator experience:

1. The acceptability score was calculated as the sum of four items rated on 0‐10 Likert scales: (1) the extent to which the simulator experience was perceived as helpful for the future realization of a safety plan, (2) the extent to which participants felt they had learned from the experience, (3) the likelihood of recommending an AI-based simulation to other professionals before implementing a safety plan, and (4) the perceived quality of the feedback received from the simulator. Higher scores indicated greater acceptability (maximum score of 40).
2. The realism score was defined as the perceived similarity of the simulator to clinical encounters. It was based on two items: (1) resemblance to a human interaction and (2) closeness to clinical experience. Higher scores indicated stronger perceived realism (maximum score of 20).
3. The challenge score was designed to capture the extent to which the simulator elicited engagement and difficulty. It included three items: (1) level of discomfort, (2) level of cognitive challenge, and (3) level of emotional challenge. Higher scores reflected a greater level of challenge experienced by participants (maximum score of 30).

### Statistical Analysis

Categorical variables are described using frequency distributions, while quantitative variables are summarized with means, SDs, and medians. Participants were categorized into 2 age groups (24‐32 years and 42‐50+ years, based on the observed age distribution) and compared on variables related to the themes of acceptability and feasibility. Age was selected as an exploratory stratification variable due to its known association with attitudes toward and adoption of digital technologies, including AI-based tools. For each variable measured before and after the simulation, individual changes were examined and tested for statistical significance using the Wilcoxon signed-rank test; the results are presented in Figure S1 in Multimedia Appendix 3. A significance threshold of *P*≤.05 was used for all statistical tests. All analyses were performed using R statistical software, version 4.3.2.

## Results

### Sample

A total of 20 mental health care professionals participated in the study (Table 1). The majority were nurses (n=18, 90%), while 2 (10%) participants were nursing assistants. Most participants (n=18, 90%) were women. The mean age was 40.75 (SD 10.41) years. Participants reported an average of 6.05 (SD 3.3; median 4.5) years of clinical experience in mental health care. Regarding prior training in safety planning, 3 (15%) participants had received no training, 6 (30%) had completed a course, and 11 (55%) had undergone a more extensive formation. None of the participants reported prior training or experience in the use of AI in psychiatry.

**Table 1.** Participant demographics.

Characteristics	Values
Gender, n	
Female	18
Male	2
Age (y), mean (SD); median (IQR)	40.75 (10.41); 44 (17.25)
Clinical experience in mental health (y), mean (SD); median (IQR)	6.05 (3.3); 4.5 (7)
Professional role, n	
Nursing assistant	2
Nurses	18
Safety plan training, n	
No training	3
Course	6
Complete formation	11
Use of AI^a^ in psychiatry	0

a AI: artificial intelligence.

### Presimulation Assessment

Before interacting with the simulator, participants generally reported high levels of confidence and preparedness regarding safety planning (Table 2). The mean perceived ability to conduct a safety plan was 7.8 (SD 2.14; median 8, IQR 3.5). Similarly, perceived competence to develop a safety plan was rated at 7.3 (SD 2.11; median 8, IQR 3.25), and the perception of having the necessary tools to complete a safety plan was 7.65 (SD 2.11; median 8, IQR 2.5). Participants also expressed a relatively high willingness to engage with patients experiencing suicidal ideation: the desire to receive patients at risk of suicide for the purpose of safety planning was rated at 7.1 (SD 2.59; median 7.5, IQR 4), while the willingness to manage patients presenting with active suicidality was slightly lower, with a mean score of 6.65 (SD 2.91; median 7.5, IQR 4.5).

**Table 2.** Presimulation and postsimulation ratings.

Questions	Ratings, mean (SD); median (IQR)
Before simulation	
To what extent do you feel able to draw up a safety plan for a patient?	7.8 (2.14); 8 (3.5)
To what extent do you feel competent to draw up a safety plan for a patient?	7.3 (2.11); 8 (3.25)
To what extent do you feel you have the necessary tools to draw up a safety plan?	7.65 (2.11); 8 (2.5)
If you had the opportunity, to what extent would you like to receive patients at risk of suicide to implement a safety plan?	7.1 (2.59); 7.5 (4)
If a patient facing active suicidality were referred to you, to what extent would you wish to manage him/her?	6.65 (2.91); 7.5 (4.5)
After simulation	
To what extent do you feel able to draw up a safety plan for a patient?	8.1 (1.59); 8 (2.25)
To what extent do you feel competent to draw up a safety plan for a patient?	7.95 (1.64); 8 (2)
To what extent do you feel you have the necessary tools to draw up a safety plan?	8.16 (1.46); 8 (2)
If you had the opportunity, to what extent would you like to receive patients at risk of suicide to implement a safety plan?	7.8 (2.14); 8 (2.5)
If a patient facing active suicidality were referred to you, to what extent would you wish to manage him/her?	7.75 (2.07); 8 (2.5)
To what extent did the simulator experience help you in the future realization of a safety plan?	7.65 (1.57); 8 (1.25)
To what extent do you feel you have learned from this experience?	7.6 (1.79); 8 (2)
Would you recommend an AI-based simulation experience to other therapists before making a safety plan?	8.3 (1.59); 9 (2.25)
What was the quality of the feedback you received?	8.35 (1.27); 8.5 (1.25)
How much discomfort did you feel?	5.21 (3.1); 5 (6)
What was the level of cognitive challenge?	6.05 (2.82); 7 (3.5)
What was the level of emotional challenge?	6.16 (2.46); 7 (4)
To what extent did you feel it resembled a human interaction?	7.55 (2.16); 7.5 (2.5)
How close is this experience to clinical experience?	7.55 (1.96); 7.5 (3)
Acceptability composite score	31.9 (5.3); 32 (7)
Realism composite score	15.1 (4.1); 15 (5.25)
Challenge composite score	17 (8); 18 (12.5)

### Postsimulation Assessment

**Figure 1.**
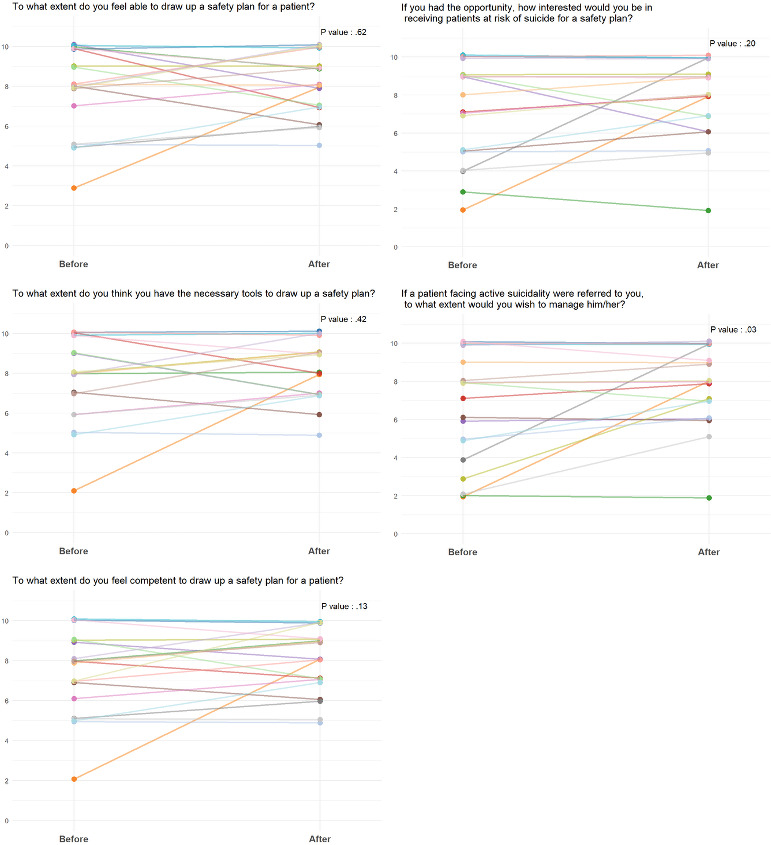
Differences before and after simulation regarding realization of safety plan.

After interacting with the simulator, participants generally reported positive experiences and perceived benefits (Figure 1; Table 2). The perceived usefulness of the simulator for the future realization of safety plans was rated at 7.65 (SD 1.57; median 8, IQR 1.25). Participants also felt that they had learned from the experience (mean 7.6, SD 1.79; median 8, IQR 2) and expressed a strong willingness to recommend an AI-based simulation to other professionals before conducting safety planning (mean 8.3, SD 1.59; median 9, IQR 2.25).

Participants’ self-perceived ability to conduct a safety plan slightly increased compared to presimulation ratings, with a mean of 8.1 (SD 1.59; median 8, IQR 2.25). Similarly, perceived competence was rated at 7.95 (SD 1.64; median 8, IQR 2), and the perception of having the necessary tools to develop a safety plan was rated at 8.16 (SD 1.46; median 8, IQR 2). Willingness to care for patients experiencing suicidal ideation also increased modestly, with participants reporting an interest in receiving patients at risk of suicide (mean 7.8, SD 2.14; median 8, IQR 2.5) and a willingness to manage patients with active suicidality (mean 7.75, SD 2.07; median 8, IQR 2.5). However, no significant differences were observed between presimulation and postsimulation ratings except regarding the willingness to actively manage patients experiencing suicidal ideation (*P=*.03)*.*

In terms of specific aspects of the simulation, participants rated the quality of feedback received highly (mean 8.35, SD 1.27; median 8.5, IQR 1.25). The simulation was perceived as moderately challenging: discomfort was rated at 5.21 (SD 3.1; median 5, IQR 6), cognitive challenge at 6.05 (SD 2.82; median 7, IQR 3.5), and emotional challenge at 6.16 (SD 2.46; median 7, IQR 4). Participants indicated that the interaction resembled a human exchange (mean 7.55, SD 2.16; median 7.5, IQR 2.5) and was close to real clinical experience (mean 7.55, SD 1.96; median 7.5, IQR 3).

Finally, the composite scores reflected these overall impressions: the acceptability score averaged 31.9 out of 40 (SD 5.3; median 32, IQR 7), the realism score averaged 15.1 out of 20 (SD 4.1; median 15, IQR 5.25), and the challenge score averaged 17.0 out of 30 (SD 8; median 18, IQR 12.5).

### Open-Ended Responses

The analysis of the open-ended questions provided additional insights into participants’ experiences with the simulator (Table 3). Regarding overall impressions, most participants described the learning experience as positive (n=12) or positive with some reservations (n=3), while 3 reported a negative experience and 2 did not answer. Suggestions for improvement primarily concerned the need for more time with the simulator (n=6), while a smaller number (n=2) mentioned increasing interactivity or providing alternative formats, such as oral feedback (n=2). Concerning advantages, participants highlighted opportunities for training and self-improvement (n=4), innovation (n=3), and realism (n=2), while 6 mentioned other individual benefits (eg, autonomy to train, reflection on practice). Potential risks were rarely identified: the majority reported none (n=16), while only 4 mentioned possible concerns (eg, being replaced by AI).

**Table 3.** Answer from participants to open questions.

Questions	Number of respondents
How did you experience learning with an AI^a^-based simulator?	
Negative	3
No answer	2
Positive	12
Positive with reservations	3
What would you suggest to improve the experience?	
Other^b^	2
Oral experience	2
More time	6
Nothing or no answer	10
What advantages do you see in this type of training?	
Other^c^	6
Training or improvement	4
Innovative	3
No answer	5
Realism	2
What are the risks or concerns associated with this type of training?	
None or no answer	16
Risk mentioned^d^	4

a AI: artificial intelligence.

b “More practice”; “To have a button to retrieve the conversation.”

c “The precision of information”; “Think about things we wouldn’t think about”; “Better than written simulations”; “Autonomy to train yourself”; “Reflection on practice”; “Quick awareness of the potential difficulty of an interview, a little time to think about the answer (writing it down rather than responding off the cuff).”

d “Frustration”; “Be replaced by AI; “Time”; “To develop our answers poorly?”

Representative comments included: “Better than usual simulations” and “Quick awareness of the potential difficulty of an interview.” These responses suggest that the simulator was generally perceived as a useful and innovative training tool, with the main limitation being the short amount of time available for practice.

### Acceptability and Feasibility Differences Among Age Groups

We further examined differences in perceptions of the simulator according to age group (24‐32 years vs 42‐50+ years; Table 4). Overall, younger participants tended to provide higher ratings than older participants on several dimensions. Significant differences were observed for the likelihood of recommending the AI-based simulator to other professionals (*P=*.03), the perceived resemblance to a human interaction (*P=*.02), and the perceived closeness to clinical experience (*P=*.05). Younger participants also provided higher ratings of feedback quality (*P=*.05) and reported stronger learning from the experience (*P=*.05) despite a same level of training than the older one at baseline. At the composite score level, younger participants scored significantly higher on acceptability (*P=*.04) and realism (*P=*.03), whereas no differences were observed for the challenge score.

**Table 4.** Acceptability and feasibility differences among age groups.

Variable	Age group (24‐32 y; n=7)	Age group (42‐50+ y; n=13)	*P* value^a^
To what extent has the simulator experience helped you in the future realization of a safety plan?, mean (SD); median (IQR)	8.29 (2.06); 9.00 (3.00)	7.31 (1.18); 8.00 (1.00)	.20
To what extent do you feel you have learned from this experience?, mean (SD); median (IQR)	8.71 (1.38); 9.00 (2.50)	7.00 (1.73); 7.00 (1.00)	.05
How would you rate the quality of the feedback you received?, mean (SD); median (IQR)	9.14 (0.90); 9.00 (1.50)	7.92 (1.26); 8.00 (2.00)	.046
Would you recommend an AI^b^-based simulation experience to other therapists before making a safety plan?, mean (SD); median (IQR)	9.29 (1.11); 10.00 (1.00)	7.77 (1.59); 8.00 (2.00)	.03
How much discomfort did you feel?, mean (SD); median (IQR)	4.29 (3.99); 2.00 (6.50)	5.75 (2.49); 5.50 (4.00)	.30
What was the level of cognitive challenge?, mean (SD); median (IQR)	4.57 (3.87); 2.00 (7.00)	6.85 (1.77); 7.00 (2.00)	.30
What was the level of emotional challenge?, mean (SD); median (IQR)	5.29 (2.98); 4.00 (5.00)	6.67 (2.06); 7.00 (2.25)	.40
To what extent did it feel like human interaction?, mean (SD); median (IQR)	9.00 (1.41); 10.00 (2.00)	6.77 (2.13); 7.00 (2.00)	.02
How close is this experience to a clinical experience?, mean (SD); median (IQR)	8.71 (1.50); 9.00 (2.00)	6.92 (1.93); 7.00 (3.00)	.05
Would you recommend an AI-based simulation experience to other therapists before making a safety plan? n (%)			.20
Below median	1 (14)	7 (54)	
Above or equal to median	6 (86)	6 (46)	
Acceptability score, mean (SD); median (IQR)	35.4 (5.0); 35.0 (7.0)	30.0 (4.5); 29.0 (6.0)	.04
Realism score, mean (SD); median (IQR)	17.7 (2.8); 18.0 (3.5)	13.7 (4.0); 14.0 (5.0)	.03
Challenge composite score, mean (SD); median (IQR)	14 (10); 11 (17)	19 (6); 18 (7)	.30

a Wilcoxon rank sum test; Fisher exact test

b AI: artificial intelligence.

### Sensitivity Analysis

**Figure 2.**
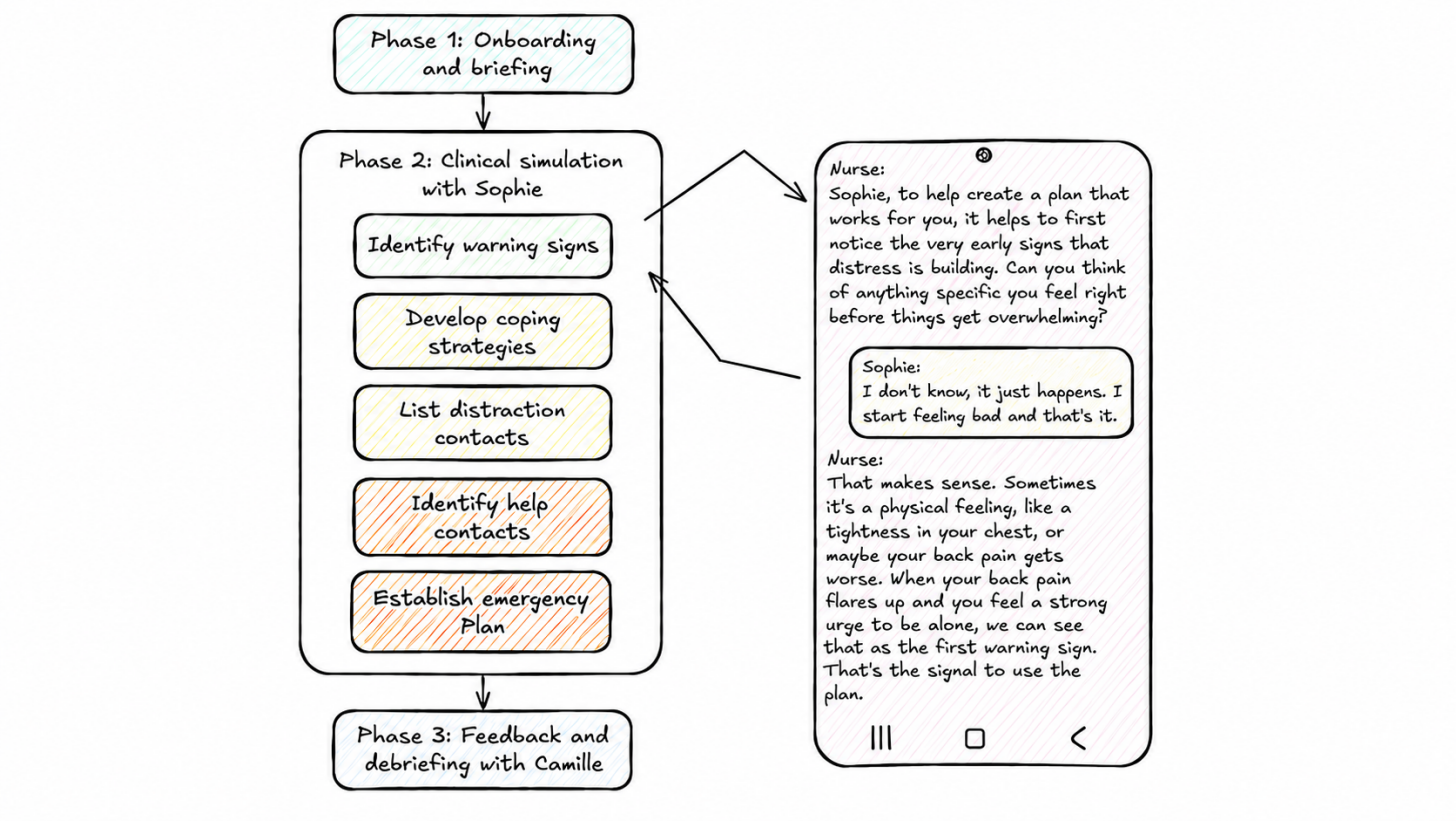
Generative artificial intelligence (GenAI) simulator.

A sensitivity analysis was conducted by excluding the 2 nursing assistants from the sample, leaving only nurses (n=18; Table S1 in Multimedia Appendix 2, Figure 2, Table 4). The results were highly similar to those obtained with the full sample.

## Discussion

This pilot study is, to our knowledge, the first to evaluate the acceptability and feasibility of a GenAI-based simulator for training mental health care professionals in safety planning. Overall, participants reported positive experiences with the tool, with mean scores approximately 7 and median scores approximately 8 across most items related to usefulness, acceptability, and perceived benefits of the simulator.

Our findings indicate a high level of acceptability for the GenAI-based simulator. Scores observed in our study compare favorably with those reported in other studies assessing the acceptability of digital training interventions [16,20,21], suggesting that the tool was well received by experienced professionals. This is particularly noteworthy in the context of nursing education, where recent literature has emphasized the potential of AI-based chatbots, such as ChatGPT, to enhance self-paced learning and provide problem-based practice [22]. In psychiatry, however, the integration of AI technologies has been slower than in other specialties, partly due to skepticism or reluctance among mental health professionals, particularly regarding their use in direct patient care [23]. In contrast, AI-based tools designed to support clinician training may be perceived as more acceptable, as they do not replace clinical judgment but rather complement it. Emerging evidence suggests that when introduced in a transparent and supportive manner, mental health practitioners are open to adopting AI-enabled tools for training purposes [24,25]. The fact that our sample consisted almost exclusively of professionals already trained in safety planning and working in specialized services for patients experiencing suicidal ideation further strengthens the relevance of our findings. While this suggests that the simulator is acceptable even among experienced providers, its perceived usefulness among less experienced professionals remains to be determined, as their needs and expectations may differ.

Open-ended feedback further supports the acceptability and feasibility signaled by the quantitative data. Most participants described the experience positively, highlighting the perceived usefulness for training, innovation, and realism. The most frequent suggestion was simply “more time” with the tool, which aligns with our procedural constraint (20 min) and suggests that longer or repeated sessions could unlock greater perceived benefits. Participants valued the feedback component, and some comments advocated for enhanced interactivity and practical affordances (eg, oral feedback options), pointing to concrete, low-cost design improvements that could increase engagement. Interestingly, very few risks were raised, which, together with only moderate reported discomfort or challenges, suggests that the simulator can be confidently integrated within standard educational oversight. Nevertheless, although only 1 participant explicitly mentioned the fear of being “replaced by AI,” this concern deserves attention given its prominence in the literature [26,27]. Job-replacement anxiety is relatively common among health care professionals and may negatively affect willingness to adopt AI-based tools [27]. This underlines the need to frame such technologies not as substitutes for clinical expertise, but as copilots designed to support decision-making, alleviate workload, and enhance the quality of care.

The simulator did not significantly change participants’ self-rated ability, competence, or perceived resources to conduct a safety plan. This result was expected, as the primary aim of the study was not to improve participants’ capacity to perform safety planning. Indeed, the sample consisted of highly experienced professionals, with an average of 6 years working in specialized psychiatric settings and accustomed to conducting safety plans daily. Moreover, participants only had a single 20-minute exposure to the simulator and interacted with just 1 patient vignette, whereas the tool is intended to be used repeatedly with a wide variety of patient profiles. Nevertheless, it is noteworthy that even under these limited conditions, participants reported a greater willingness to actively care for patients experiencing suicidal ideation, which suggests a potential benefit in terms of enhancing clinicians’ confidence and comfort. This finding is encouraging, as repeated use of the simulator may further strengthen such effects. Importantly, no decrease was observed in participants’ self-perceived abilities after the simulation. One might have anticipated that receiving automated feedback highlighting errors or areas for improvement could reduce self-confidence; however, this was not the case, which is reassuring for the acceptability of the tool.

Age-related differences also emerged in our study, with younger participants reporting higher levels of acceptability and realism than their older colleagues. This pattern is consistent with the findings from other fields of medicine showing that younger health care professionals and medical students tend to hold more positive attitudes toward AI than older practitioners, who are often more cautious or reluctant to adopt new technologies [26,28,29]. Such generational differences may reflect greater exposure to digital technologies among younger cohorts, as well as their expectation that AI will become an integral part of future clinical practice. By contrast, older professionals may be more influenced by concerns related to the reliability, ethical implications, or impact of AI on clinical roles. These findings underscore the importance of addressing potential gaps in digital literacy and technology readiness among more senior clinicians. At the same time, they highlight the need to systematically integrate AI-related training into medical and nursing curricula. Indeed, recent research emphasizes that early, formal education on the opportunities and limitations of AI in health care is essential to foster informed, critical, and confident use of these tools among future professionals [22,28]. Implementing structured AI teaching modules in medical schools and nursing programs could therefore play a decisive role in preparing the next generation of clinicians to adopt and effectively use such technologies in their practice.

Nevertheless, the integration of GenAI into psychiatric training raises several ethical challenges that must be carefully addressed [13,30-32]. While tools such as our simulator hold promise for democratizing access to high-quality training and reducing barriers to continued professional education, concerns persist regarding data privacy, algorithmic bias, and professional responsibility. As highlighted by Blease and Torous [31], large language models can generate inaccurate or biased outputs, raising risks if their content is not properly supervised. Recent exploratory work in psychiatry emphasizes the importance of structured ethical education and continuous oversight to guide the safe integration of AI in practice. Finally, broader debates on the “democratization” of AI in mental health stress the need for transparency, inclusivity, and governance mechanisms that protect patient rights and ensure equitable access [30]. Together, these considerations underline the necessity of implementing such tools within robust ethical frameworks, coupled with ongoing training, supervision, and clear regulatory guidance.

This study provides one of the first explorations of the feasibility and acceptability of a GenAI-based simulator for training mental health professionals in safety planning. Its strengths include the use of a specialized sample of nurses and nursing assistants routinely working with patients experiencing suicidal ideation, as well as the combination of quantitative and qualitative data to capture participants’ perceptions in detail. Nevertheless, several limitations should be acknowledged. The small sample size and the single-center design limit the generalizability of our findings, and the short interaction with the simulator (20 minutes, based on a single vignette) prevented the assessment of repeated practice effects. Furthermore, the study was conducted exclusively among nurses and nursing assistants, the majority of whom were women, which may further restrict the applicability of the results to other professional groups. Moreover, participants reported high baseline levels of confidence and preparedness, which may have introduced ceiling effects and limited the ability to detect changes following the simulation. In addition, because participants were already experienced in suicide prevention and safety planning, our results may underestimate or overestimate the potential benefits for less experienced professionals. Then, detailed process metrics (eg, interaction duration variability, number of exchanges, and objective assessment of AI-generated feedback quality) were not collected, as this was beyond the scope of this pilot study focused on feasibility and acceptability. In addition, this study did not evaluate the clinical accuracy or appropriateness of the AI-generated dialog, or the quality of the safety plans produced. This was not the primary objective of this pilot study, which focused on assessing feasibility and acceptability. Future studies should specifically address these aspects using more rigorous designs, including objective evaluation of safety plan quality and clinical outcomes. In particular, we plan to perform a randomized controlled trial to compare standard safety planning training with standard training plus regular practice using the GenAI-based simulator. Such a trial would incorporate a variety of patient vignettes with different clinical profiles (eg, varying diagnoses, levels of resistance, or cultural backgrounds) to test the training’s effectiveness across a range of challenging clinical scenarios. This design would allow evaluation not only of professionals’ self-confidence and competence but also of the objective quality of the safety plans produced in clinical practice. Importantly, it would also enable the examination of patient outcomes, such as psychiatric readmissions, suicide attempts, or suicidal ideation, to determine whether improvements in training translate into measurable reductions in suicidal behaviors. Finally, the current version of the simulator is entirely text-based, relying on written exchanges. Future developments could include voice-based interactions, allowing practitioners to speak naturally as they would with a patient, or even the integration of a virtual avatar to further enhance realism and immersion. Several other improvements to the simulator could be implemented prior to large-scale evaluation. These include increasing the diversity of clinical vignettes, enhancing interactivity based on user feedback (eg, more dynamic responses or alternative feedback formats), and refining the feedback system to further align with clinical training standards. Extending the duration and frequency of training sessions may also improve learning effects.

This pilot study demonstrates that a GenAI-based simulator for safety planning is highly acceptable to mental health professionals specializing in the care of patients experiencing suicidal ideation, highlighting its promise as an innovative training tool. While preliminary, these findings suggest that integrating AI into mental health education could substantially enhance training opportunities and professional preparedness. The implementation of AI-assisted training represents a promising step toward the future of psychiatry, where digital tools can complement clinical expertise to strengthen both provider confidence and patient care.

## Supplementary material

10.2196/88440 Multimedia Appendix 1 Technical implementation of the simulator, including model selection, platform deployment, and prompt engineering strategies.

10.2196/88440 Multimedia Appendix 2 Participant demographics, presimulation and postsimulation ratings, and composite scores without nursing assistant.

10.2196/88440 Multimedia Appendix 3 Differences before and after simulation on capacity to realize safety plan without nursing assistant.

10.2196/88440 Multimedia Appendix 4 Acceptability and feasibility differences among age groups without nursing assistant.

## Abbreviations

AI: artificial intelligence
GenAI: generative artificial intelligence
