# Supplementary material for: Use of a Conversational Agent for Training Mental Health Professionals in Suicide Safety Planning: Pilot Feasibility and Acceptability Study
Source: JMIR Ment Health. 2026 Jun 30;13:e88440. doi: 10.2196/88440 (PMC13317675; doi:10.2196/88440)
Supplement: Multimedia Appendix 1 [file mental-v13-e88440-s001.docx]

*Appendix A*

*Technical Implementation of the Simulator*

## Technological Framework: Model and Platform Selection

The choice of the Claude 3.5 Sonnet V2 model was deliberate, following a comparative analysis of its performance against other models available at the time of development. This model was selected for several critical advantages essential for this complex simulation. First, it demonstrates a strong capability to maintain persona and stage consistency throughout a prolonged interaction, a vital feature for the simulator’s dual-stage structure. Second, it is known for a relatively low hallucination rate, high response speed, and robust linguistic capabilities. This reliability is particularly crucial during the feedback stage, where the model is required to analyze the conversation accurately, without omitting important events that occurred or inventing (hallucinating) events that did not. The model offers an optimal combination of these capabilities, making it particularly suitable for running a complex simulation based entirely on a single prompt.

The simulator was deployed to participants using the PMFM AI platform. This is a platform that enables developers and researchers to make AI-based bots accessible to end-users. The platform allows for the input of a prompt, the selection of the desired language model (in this case, Claude 3.5 Sonnet V2), and the addition of knowledge files and appendices using Retrieval-Augmented Generation (RAG). Once the bot is configured, the system generates a unique link that is sent to participants. This link allows them to conduct a conversation with the simulator without message limits and to receive the full feedback at its conclusion. Additionally, the platform allows for the storage of all conversation transcripts for research and analysis purposes, subject to participant consent. Specifically, the platform includes built-in functionality for saving and exporting conversation transcripts, including the safety plan content and the automated feedback. However, in the present study, the simulator was configured to ensure participant anonymity: conversations were conducted without linkage to individual participant identifiers, and therefore the research team could not collect conversation data on a per-participant basis. Participants could export their own conversation by copying and pasting the content into an external document, but no dedicated download feature was available within the platform interface during the study. Future research could leverage the platform’s export capabilities in a non-anonymous configuration, enabling direct scoring of finalized safety plans and conversation transcripts for performance evaluation.

## Prompt Engineering and Behavioral Logic

The simulator’s clinical framework is grounded in the Safety Planning Intervention (SPI) [6]. The five-step structure (warning signs, internal coping strategies, social contacts for distraction, seeking help, and means safety) serves as the backbone of both the simulation interaction and the automated feedback evaluation. This framework was provided to the model as one of two foundational appendices within the core prompt, ensuring structured clinical guidance throughout the entire interaction. Participants constructed the safety plan collaboratively within the text-based conversation, with no external template used. The resulting plan was contained within the conversation transcript; the platform’s transcript storage and export capabilities were not used in this study due to the anonymous configuration employed to protect participant privacy (as described in the platform deployment section above).

The simulator’s dynamic and realistic behavior is achieved through several advanced prompt engineering techniques:

### High-Fidelity Persona Synthesis

The persona of “Sophie” is not a generic patient but a deeply specified character synthesized from the clinical vignette in Appendix B. Her profile includes a diagnosis of bipolar disorder, treatment with lithium salts, and a history of a previous suicide attempt. The prompt operationalizes her psychosocial stressors, including significant social isolation (deceased mother, estranged father), unemployment, financial difficulties, and the acute crisis of her ex-partner seeking to relocate with their 8-year-old son, Mathéo. Her primary protective factor, her son, is also explicitly coded as a key motivational element.

### Dynamic Interaction Logic and Conditional Triggers

The prompt moves beyond static role-playing by implementing a set of conditional rules that govern Sophie’s emotional reactivity. This logic dictates that she will exhibit increased resistance, anger, or distrust in response to specific conversational triggers, such as direct questions about her ex-partner or a perceived rushed or impersonal approach from the nurse. Conversely, the prompt instructs her to become more cooperative when the participant demonstrates patience, validates her feelings, and shows genuine interest in her well-being, particularly concerning her son. This creates a more challenging and realistic simulation where the participant’s therapeutic alliance skills are actively tested.

### Instructional Scaffolding for Feedback

The “Camille” persona’s feedback is not left to the model’s general knowledge. The prompt provides a rigorous scaffolding structure mandating that the analysis adhere strictly to an 11-point evaluation rubric organized across four domains:

*(a) Contextual framing* (2 criteria): (1) identification of the report topic; and (2) documentation of the number of interaction messages, providing a quantitative measure of engagement.

*(b) Process quality* (2 criteria): (3) a detailed analysis of the conversation flow, key turning points, and overall interaction dynamics, noting specific moments where clinical opportunities were taken or missed; and (4) a critical assessment of the therapeutic relationship, evaluating the nurse’s ability to establish empathic connection, warmth, and appropriate communication despite patient resistance, with reference to specific examples from the conversation.

*(c) Safety plan content* (5 criteria, one per step): (5) identification of warning signs — how effectively the nurse helped the patient recognize personal indicators of increasing distress; (6) personal coping strategies — the nurse’s ability to help develop concrete, practical self-management techniques; (7) distraction contacts — how thoroughly the nurse helped identify people and places that provide thought distraction; (8) help-seeking contacts — how the nurse guided creation of a specific support list with names and phone numbers; and (9) emergency planning — the nurse’s approach to identifying emergency contacts, securing the environment, and reinforcing the main protective factor.

*(d) Clinical synthesis* (2 criteria): (10) integration — how well all components were combined into a coherent, usable tool, including summarization, verification of understanding, and identification of potential weaknesses; and (11) overall summary — an assessment of strengths and weaknesses, an overall score (1–10), and three specific, actionable recommendations for professional development.

A detailed scoring system (1–10) is embedded in the prompt with explicit criteria across five performance levels: scores of 9–10 reflect clinical excellence with exceptional therapeutic alliance, comprehensive personalized planning, and effective resistance management; scores of 7–8 indicate good clinical skill with meaningful therapeutic relationship and coverage of most required areas; scores of 5–6 represent basic skill with significant gaps and missed opportunities; scores of 3–4 indicate deficient skill with superficial relationship and substantial misses; and scores of 1–2 correspond to severe clinical failures with fundamental misunderstanding of safety planning principles. If the safety plan was only partially completed or the simulation ended early, the score is capped at 6 regardless of process quality. This ensures that the feedback is standardized, critical, and educationally potent.

To ensure the accuracy of the automated feedback, the research team conducted iterative testing during the design phase. Multiple simulated conversations were run, and the AI-generated feedback was reviewed against expert clinical judgment. Prompt instructions were progressively refined based on identified discrepancies — for example, ensuring the model did not omit critical events from the conversation transcript or generate (hallucinate) events that did not occur. The validity of GenAI-generated feedback in a comparable suicide prevention training context has been demonstrated in a parallel study by our team [Haber et al., 2025], which showed strong alignment between AI-generated and expert-rated skill assessments. Nevertheless, as this is among the first applications of GenAI-generated feedback in clinical simulation training, further validation studies are needed to rigorously assess the feedback’s accuracy, depth, and sensitivity to potential cultural and gender biases across diverse trainee populations and clinical contexts.

### Finite State Machine (FSM) Workflow

The prompt architecture functions as a finite state machine, guiding the AI and the participant through a non-deviating sequence of stages. The system progresses methodically from onboarding and briefing to the simulation itself, to a clean transition between personas, and finally to a structured feedback and debriefing phase. This prevents conversational drift and ensures that every participant experiences the full, intended educational workflow.
