# Supplementary material for: Use of a Conversational Agent for Training Mental Health Professionals in Suicide Safety Planning: Pilot Feasibility and Acceptability Study
Source: JMIR Ment Health. 2026 Jun 30;13:e88440. doi: 10.2196/88440 (PMC13317675; doi:10.2196/88440)
Supplement: Multimedia Appendix 2 [file mental-v13-e88440-s002.docx]

Supplementary table 1: Participant demographics, pre- and post-simulation ratings, and composite scores without nursing assistant

| **Question** | **Categorical levels** | **Mean [sd]** | **Median** |
| --- | --- | --- | --- |
| Gender | Female: 16; Male: 2 |  |  |
| Age | 24-32: 7; 42-50+: 11 | 39.78 [10.37] |  |
| Years of clinical experience in mental health |  | 5.89 [3.34] | 4 |
| Safety plan training | No training: 3; Course: 5; Complete formation: 10 |  |  |
| Use of AI in psychiatry | No participant concerned |  |  |
| To what extent do you feel able to draw up a safety plan for a patient? |  | 8.11 [2.03] | 8 |
| To what extent do you feel competent to draw up a safety plan for a patient? |  | 7.56 [2.06] | 8 |
| To what extent do you feel you have the necessary tools to draw up a safety plan? |  | 7.78 [2.18] | 8 |
| If you had the opportunity, to what extent would you like to receive patients at risk of suicide in order to implement a safety plan? |  | 7.44 [2.5] | 8.5 |
| If a patient facing active suicidality were referred to you, to what extent would you wish to manage him/her? |  | 7.06 [2.75] | 8 |
| To what extent did the simulator experience help you in the future realization of a safety plan? |  | 7.67 [1.64] | 8 |
| To what extent do you feel you have learned from this experience? |  | 7.56 [1.89] | 7.5 |
| Would you recommend an AI-based simulation experience to other therapists before making a safety plan? |  | 8.33 [1.64] | 9 |
| To what extent do you feel able to draw up a safety plan for a patient? |  | 8.33 [1.5] | 8.5 |
| To what extent do you feel competent to draw up a safety plan for a patient? |  | 8.22 [1.48] | 8.5 |
| To what extent do you think you have the necessary tools to draw up a safety plan? |  | 8.22 [1.48] | 8.5 |
| If you had the opportunity, how interested would you be in receiving patients at risk of suicide for a safety plan? |  | 7.83 [2.09] | 8 |
| If a patient facing active suicidality were referred to you, how would you like to manage him/her? |  | 7.78 [2.02] | 8 |
| How would you rate the quality of the feedback you received? |  | 8.44 [1.2] | 8.5 |
| How much discomfort did you feel? |  | 5.06 [3.21] | 5 |
| What was the level of cognitive challenge? |  | 5.94 [2.94] | 7 |
| What was the level of emotional challenge? |  | 6 [2.43] | 7 |
| To what extent did you feel it resembled a human interaction? |  | 7.67 [2.09] | 7.5 |
| How close is this experience to clinical experience? |  | 7.67 [1.85] | 7.5 |
| Acceptability composite score |  | 32.0 [5.5] | 32 |
| Realism composite score |  | 15.3 [3.9] | 15 |
| Challenge composite score |  | 17 [8] | 18 |
| How did you experience learning with an AI-based simulator? | Negative: 3; No answer: 2; Positive: 11; Positive with reservations: 2 |  |  |
| What would you suggest to improve the experience? | Other^1^: 2; Oral experience: 2; More time: 6; Nothing/No answer: 8 |  |  |
| What advantages do you see in this type of training? | Other^2^: 6; Training/improvement: 4; Innovative: 3; No answer: 3; Realism: 2 |  |  |
| What are the risks or concerns associated with this type of training? | None/no answer: 14; Risk mentioned: 4 |  |  |
