# Supplementary material for: Use of a Conversational Agent for Training Mental Health Professionals in Suicide Safety Planning: Pilot Feasibility and Acceptability Study
Source: JMIR Ment Health. 2026 Jun 30;13:e88440. doi: 10.2196/88440 (PMC13317675; doi:10.2196/88440)

Supplementary Figure 1: Differences before and after simulation on capacity to realize safety plan without nursing assistant


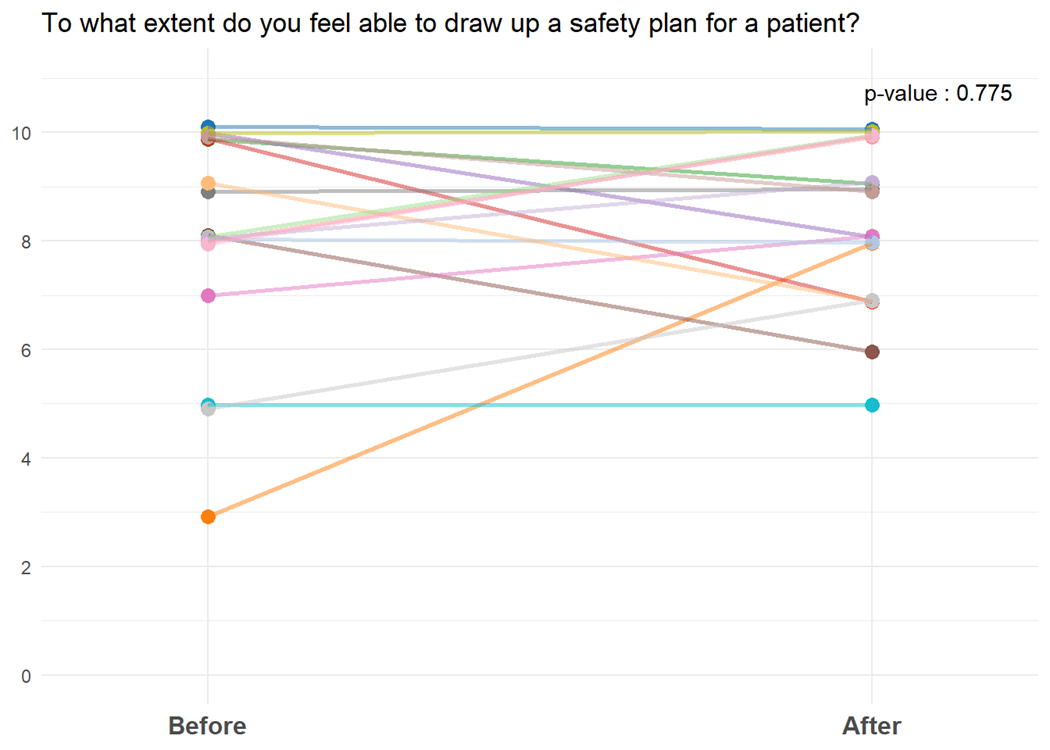

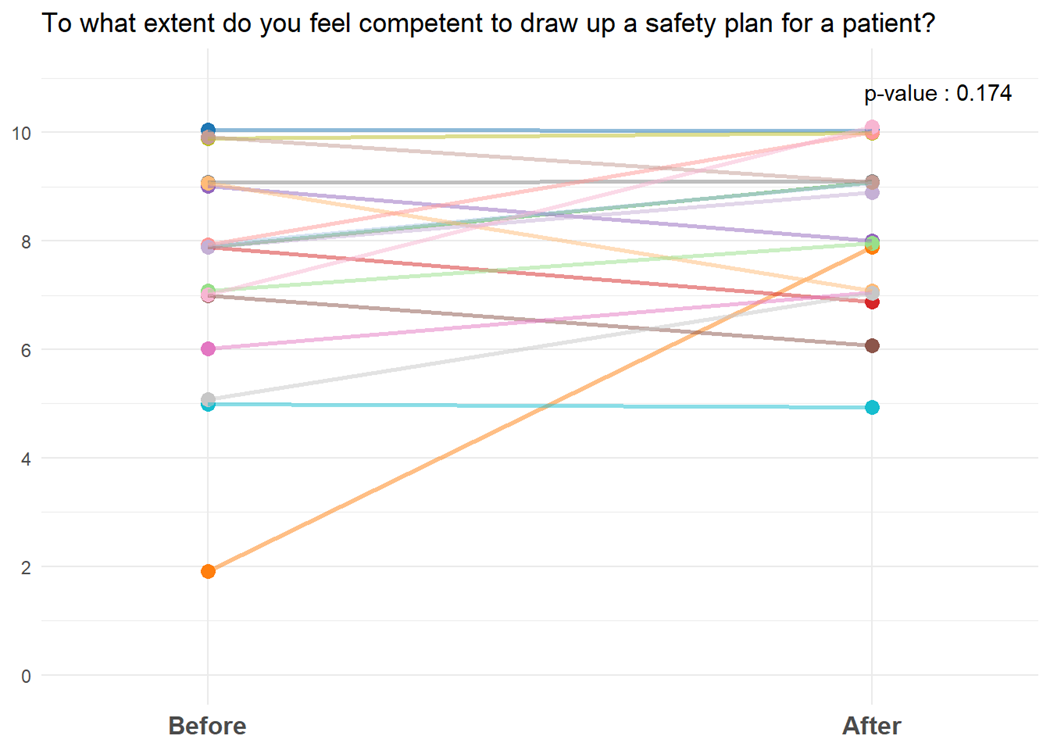

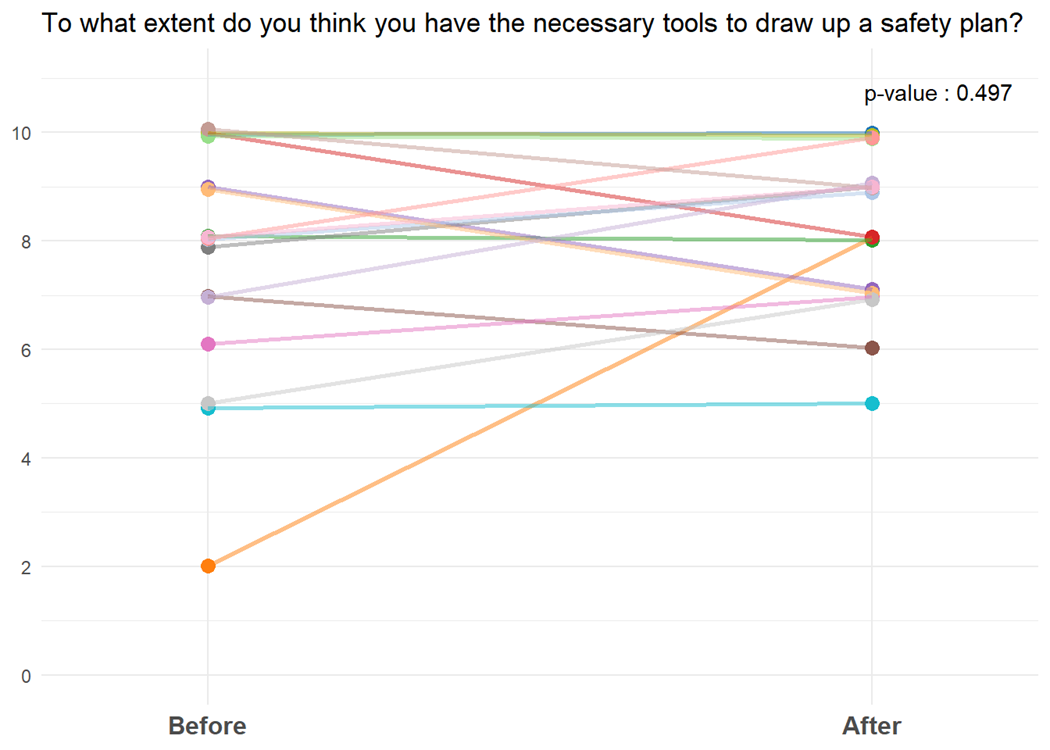

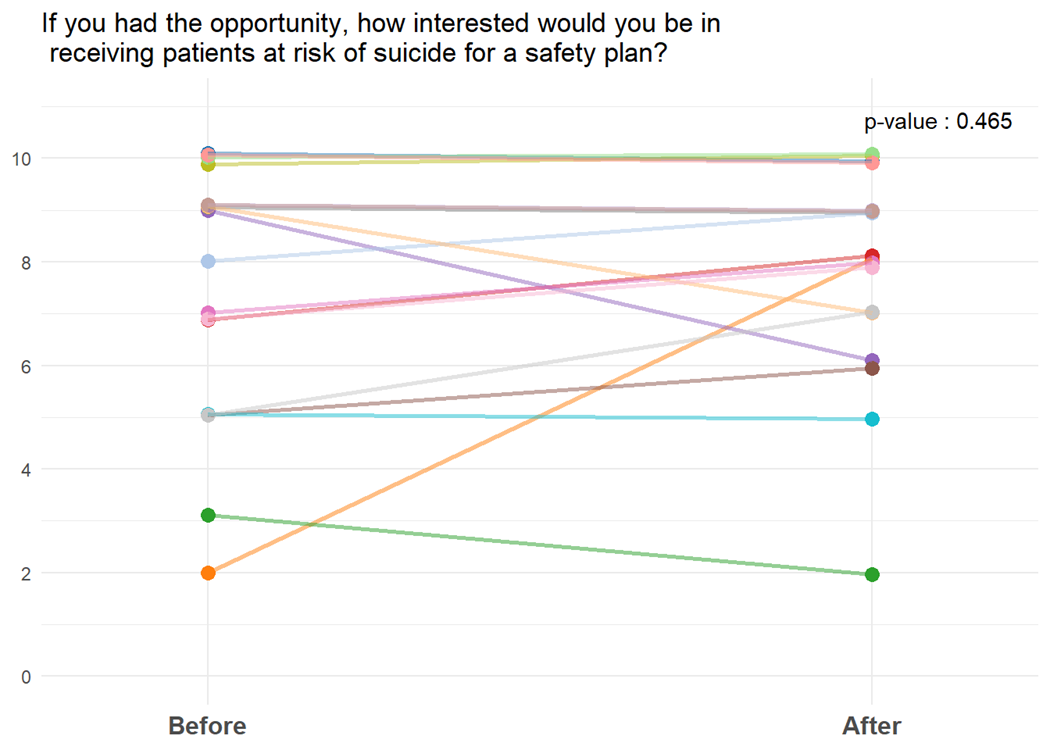

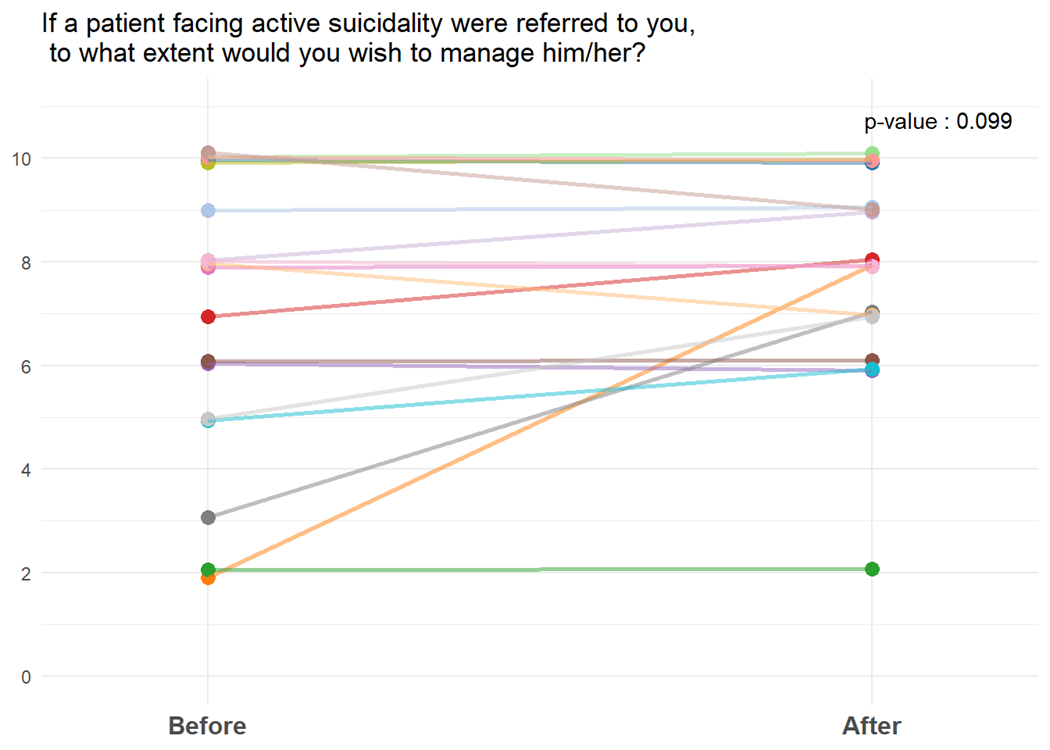

Supplement: Multimedia Appendix 3 [file mental-v13-e88440-s003.docx]
