# Supplementary material for: Use of a Conversational Agent for Training Mental Health Professionals in Suicide Safety Planning: Pilot Feasibility and Acceptability Study
Source: JMIR Ment Health. 2026 Jun 30;13:e88440. doi: 10.2196/88440 (PMC13317675; doi:10.2196/88440)
Supplement: Multimedia Appendix 4 [file mental-v13-e88440-s004.docx]

Supplementary table 2: Acceptability and feasibility differences among ages’ groups without nursing assistant

| Variable | 24-32, N = 7*^1^* | 42-50+, N = 11*^1^* | p-value*^2^* |
| --- | --- | --- | --- |
| To what extent did the simulator experience help you in the future realization of a safety plan? | 9.00, 8.29 (2.06) | 8.00, 7.27 (1.27) | 0.2 |
| To what extent do you feel you have learned from this experience? | 9.00, 8.71 (1.38) | 7.00, 6.82 (1.83) | **0.04** |
| How would you rate the quality of the feedback you received? | 9.00, 9.14 (0.90) | 8.00, 8.00 (1.18) | 0.056 |
| Would you recommend an AI-based simulation experience to other therapists before making a safety plan? | 10.00, 9.29 (1.11) | 8.00, 7.73 (1.68) | **0.04** |
| How much discomfort did you feel? | 2.00, 4.29 (3.99) | 5.50, 5.60 (2.63) | 0.4 |
| What was the level of cognitive challenge? | 2.00, 4.57 (3.87) | 7.00, 6.82 (1.89) | 0.3 |
| What was the level of emotional challenge? | 4.00, 5.29 (2.98) | 7.00, 6.45 (2.02) | 0.6 |
| To what extent did you feel it resembled a human interaction? | 10.00, 9.00 (1.41) | 7.00, 6.82 (2.04) | **0.03** |
| How close is this experience to clinical experience? | 9.00, 8.71 (1.50) | 7.00, 7.00 (1.79) | 0.059 |
| Acceptability composite score | 35.0, 35.4 (5.0) | 29.0, 29.8 (4.8) | **0.05** |
| Realism composite score | 18.0, 17.7 (2.8) | 14.0, 13.8 (3.8) | **0.04** |
| Challenge composite score | 11, 14 (10) | 18, 19 (6) | 0.3 |
| Would you recommend an AI-based simulation experience to other therapists before making a safety plan? |  |  | 0.2 |
| *Below median* | 1 (14%) | 6 (55%) |  |
| *Above or equal to median* | 6 (86%) | 5 (45%) |  |
| *^1^Median, Mean (SD); n (%)*  *^2^Wilcoxon rank sum test; Fisher's exact test* | | | |
